# Supplementary material for: Adaptation of Brucella melitensis Antimicrobial Susceptibility Testing to the ISO 20776 Standard and Validation of the Method
Source: Microorganisms. 2022 Jul 20;10(7):1470. doi: 10.3390/microorganisms10071470 (PMC9316112; doi:10.3390/microorganisms10071470)
Supplement: Supplementary file 1 [file microorganisms-10-01470-s001.zip › microorganisms-1771996-supplementary.pdf]

microorganisms

MDPI

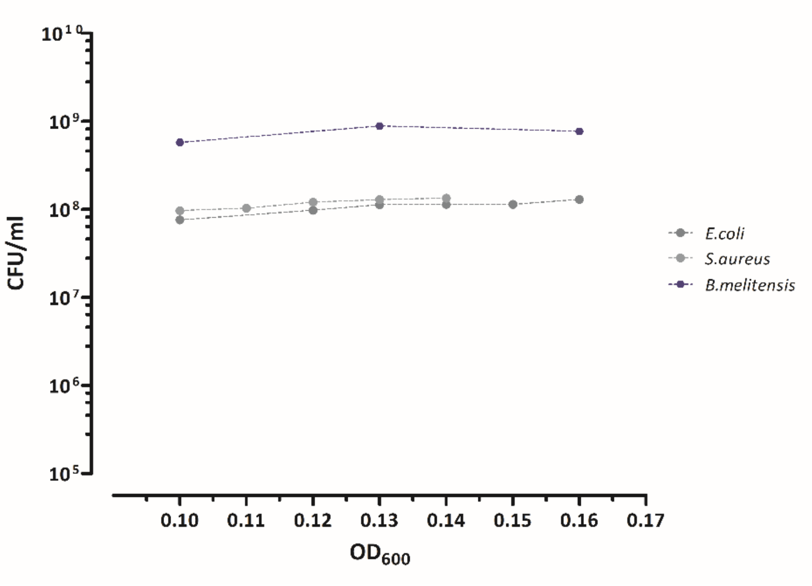

Figure S1. Determination of colony forming units (CFU) in a 0.4 to 0.6 McFarland suspension. Three bacterial strains (*E.coli* 1103, *B. melitensis* reference strain ATCC 23456 and *S. aureus*) were diluted to McFarland standards between 0.4 to 0.6 and afterwards, the corresponding OD_{600} were determined. 10-fold serial dilutions in 0.9% NaCl were made and 100 µl of each dilution were plated on Colombia agar plates. Subsequently, colonies were counted and the number of CFU/ml in the original undiluted solution was calculated.

Table S1. Comparison of five different culture media recommended for fastidious organism (BB, CAMHB, CAMHB-F, CAMHB-X and H- medium). BMD was performed in triplicates using CAMHB, CAMHB-F, CAMHB-X and H-medium and repeated six times for BB. MIC values were determined after incubation in a 5% CO_2 atmosphere for 48 h. The numbers in the table represent the range of the tested MIC values (**a**) *B. melitensis* reference strain ATCC 23456. (**b**) *B. melitensis* clinical isolate Bm150048. CIP, ciprofloxacin; LEV, levofloxacin; DOX, doxycycline; GEN, gentamicin; STR, streptomycin; SXT, trimethoprim-sulfamethoxazole; RIF, rifampicin

(a)

Antimicrobial substance	BB (mg/L)	CAMHB (mg/L)	CAMHB-X (mg/L)	CAMHB-F (mg/L)	H-Medium (mg/L)
GEN	0.5-1	0.5	0.5	0.5	0.5
STR	2-4	2	2	2	1-2
RIF	1-2	1	0.5	1-4	2
DOX	0.06-0.125	0.06	0.06	0.125-0.5	0.25-0.5
SXT	0.5-1	0.02	0.125	0.25	2
LEV	0.5	0.5	0.5	0.5	0.5
CIP	0.5	0.5	0.5	0.5	0.5

(b)

Antimicrobial substance	BB (mg/L)	CAMHB (mg/L)	CAMHB-X (mg/L)	CAMHB-F (mg/L)	H-Medium (mg/L)
GEN	0-5-2	0.25	0.25-0.5	0.5	0.5
STR	2	1	0.5-1	2	1
RIF	4	4	2	4	4
DOX	0.125-0.25	0.125	0.25	0.25	0.25-0.5
SXT	1-4	0.06	0.125-0.25	0.25-0.5	4
LEV	0.5	0.5	0.5	0.5	0.5
CIP	0.5	0.5	0.5	0.5	0.5

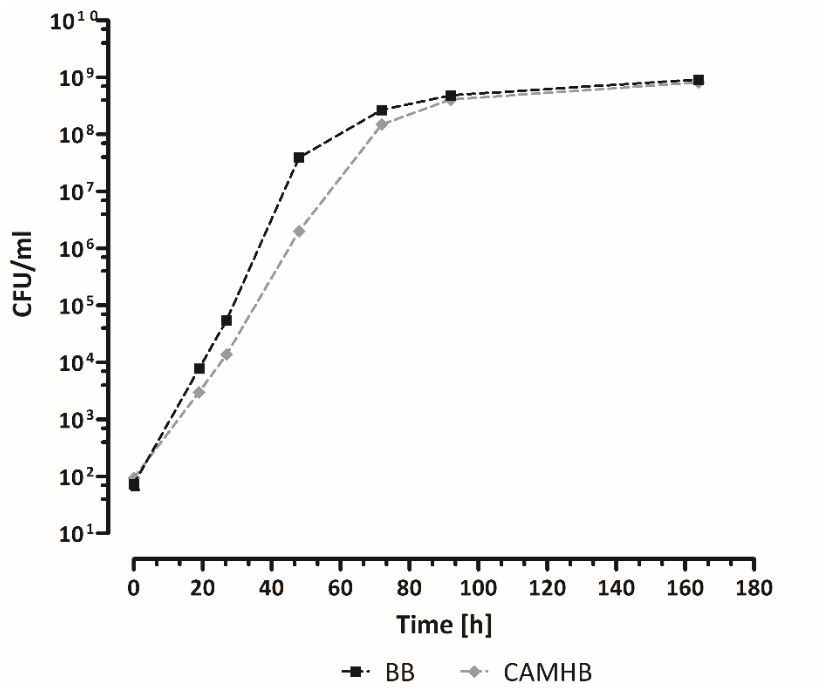

Figure S2. Growth curve of Bm150048 in two different culture media (BB, CAMHB). Each dot represents the mean value of 3 replicates. BB, *Brucella* broth; CAMHB, cation adjusted Mueller-Hinton II broth;

Table S2. Comparison of BMD and AD using two different culture media (BB, CAMHB). *B. melitensis* reference strain ATCC 23456 and *B. melitensis* clinical isolate Bm150048 were used in triplicates to compare MIC values obtained with BMD and AD when using (**a**) BB or (**b**) CAMBH. MIC values were obtained after incubation in a 5% CO_2 atmosphere for 48 h. The numbers in the table represent the MIC or range of MICs of the repeated tests. DOX, doxycycline; GEN, gentamicin; STR, streptomycin; SXT, trimethoprim-sulfamethoxazole; RIF, rifampicin

(a)

Antimicrobial substance	ATCC 23456		Bm150048	
	**BMD**	**AD**	**BMD**	**AD**
GEN (mg/L)	0.5-1	1	0.5-1	0.5-1
STR (mg/L)	2-4	4	2	2-8
RIF (mg/L)	2-4	2	4	4
DOX (mg/L)	0.06-0.125	0.25-0.5	0.25	0.25-0.5
SXT (mg/L)	0.5-1	1	0.5-1	0.5-1

(b)

Antimicrobial substance	ATCC 23456		Bm150048	
	**BMD**	**AD**	**BMD**	**AD**
GEN (mg/L)	0.5	0.25-0.5	0.25	0.25-0.5
STR (mg/L)	2	2-4	1	2-4
RIF (mg/L)	1	1	4	4
DOX (mg/L)	0.06	0.25	0.25	0.25
SXT (mg/L)	0.02	0.03-0.06	0.06	0.06-0.125

Table S3. Impact of incubation time on MIC values. BMD was performed in triplicates and MIC values were determined after incubation in 5% CO_2 for 18-64 h. The numbers in the table represent the MIC or range of MICs of the repeated tests. (**a**) CAMHB (**b**) BB. CIP, ciprofloxacin; LEV, levofloxacin; DOX, doxycycline; GEN, gentamicin; STR, streptomycin; SXT, trimethoprim-sulfamethoxazole; RIF, rifampicin

(a)

Antimicrobial substance	18 h	24 h	42 h	48 h	64 h
GEN (mg/L)	<0.016	<0.016	0.25	0.25	0.25
STR (mg/L)	<0.125	<0.125	0.5	1	1
RIF (mg/L)	<0.25	<0.25	2-4	4	4
DOX (mg/L)	<0.016	<0.016	0.125	0.125	0.125-0.25
SXT (mg/L)	<0.008	<0.008	0.031	0.031	0.062
LEV (mg/L)	<0.004	<0.004	0.5	0.5	0.5
CIP (mg/L)	<0.004	<0.004	0.5	0.5	0.5

(b)

Antimicrobial substance	18 h	24 h	42 h	48 h	64 h
GEN (mg/L)	<0.016	0.25	0.5	0.5	0.5
STR (mg/L)	<0.125	0.5-1	2	2	2
RIF (mg/L)	<0.25	2	2-4	4	4
DOX (mg/L)	<0.016	0.0625	0.125	0.125	0.25
SXT (mg/L)	<0.008	1	1-2	1-2	1-2
LEV (mg/L)	<0.004	0.25-0.5	0.5	0.5	0.5
CIP (mg/L)	<0.004	0.5	0.5	0.5	0.5

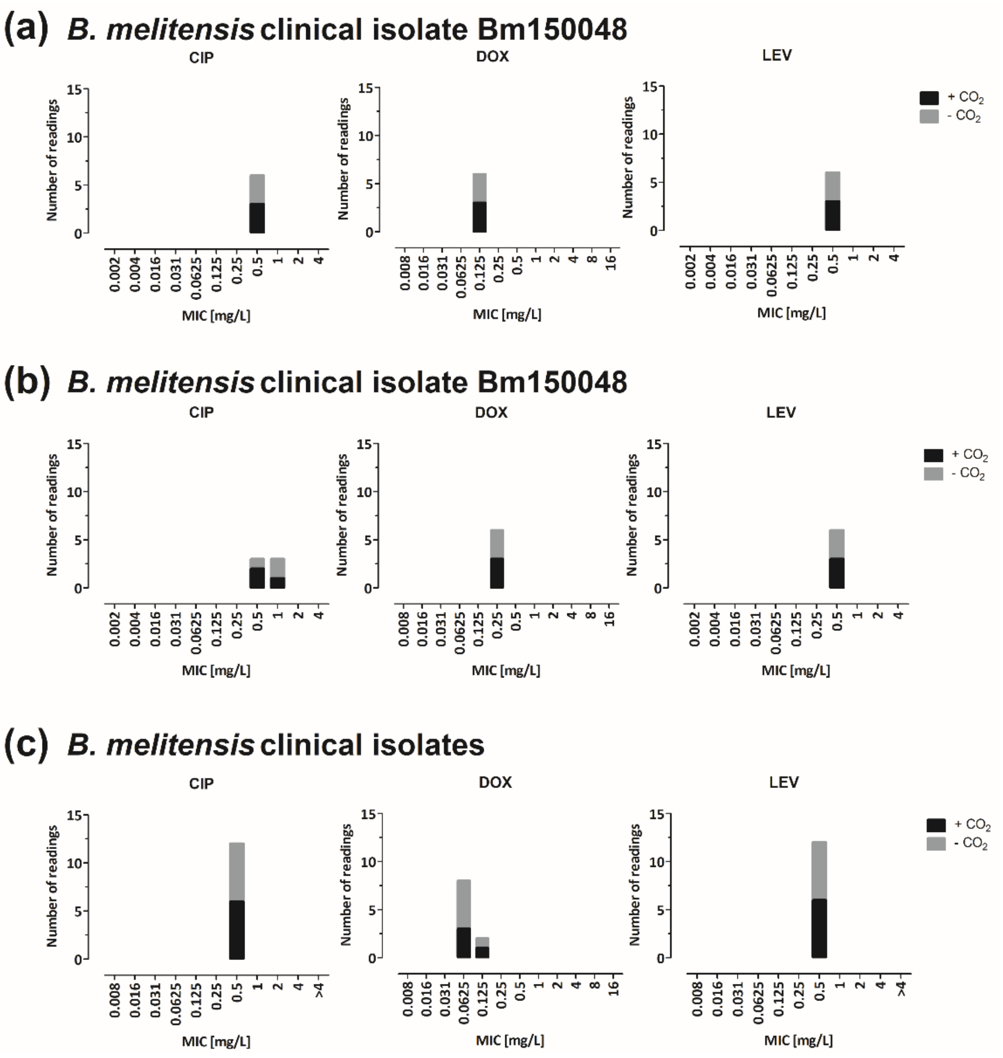

Figure S3. Impact of the cultivation atmosphere on MIC values. MIC values were determined in triplicates (**a, b**) or once (**c**) after incubation at 36 ±1°C with or without 5% CO_2 for 48 h. CAMHB was used as culture medium. (a) Reference strain ATCC 23456. (b) Clinical isolate Bm150048. (c) Six clinical isolates. CIP, ciprofloxacin; LEV, levofloxacin; DOX, doxycycline;
